# *In Vitro* Evaluation of Genotoxic Effects under Magnetic Resonant Coupling Wireless Power Transfer

**DOI:** 10.3390/ijerph120403853

**Published:** 2015-04-07

**Authors:** Kohei Mizuno, Naoki Shinohara, Junji Miyakoshi

**Affiliations:** Laboratory of Applied Radio Engineering for Humanosphere, Research Institute for Sustainable Humanosphere, Kyoto University, Gokasho, Uji, Kyoto 611-0011, Japan; E-Mails: shino@rish.kyoto-u.ac.jp (N.S.); miyakoshi@rish.kyoto-u.ac.jp (J.M.)

**Keywords:** wireless power transfer, magnetic resonant coupling, cell growth, cell cycle distribution, comet assay, micronucleus formation, HPRT gene mutation, human fibroblast cells, 12.5-MHz resonant frequency

## Abstract

Wireless power transfer (WPT) technology using the resonant coupling phenomenon has been widely studied, but there are very few studies concerning the possible relationship between WPT exposure and human health. In this study, we investigated whether exposure to magnetic resonant coupling WPT has genotoxic effects on WI38VA13 subcloned 2RA human fibroblast cells. WPT exposure was performed using a helical coil-based exposure system designed to transfer power with 85.4% efficiency at a 12.5-MHz resonant frequency. The magnetic field at the positions of the cell culture dishes is approximately twice the reference level for occupational exposure as stated in the International Commission on Non-Ionizing Radiation Protection (ICNIRP) guidelines. The specific absorption rate at the positions of the cell culture dishes matches the respective reference levels stated in the ICNIRP guidelines. For assessment of genotoxicity, we studied cell growth, cell cycle distribution, DNA strand breaks using the comet assay, micronucleus formation, and hypoxanthine-guanine phosphoribosyltransferase (HPRT) gene mutation, and did not detect any significant effects between the WPT-exposed cells and control cells. Our results suggest that WPT exposure under the conditions of the ICNIRP guidelines does not cause detectable cellular genotoxicity.

## 1. Introduction

There is public concern regarding the potential health risks of technologies using electromagnetic fields (EMFs). Research into possible relationships between exposure to EMFs and human health is very important. The World Health Organization has assessed the health risks produced by EMFs in the frequency range 0–300 GHz, and has published Environmental Health Criteria monographs to provide critical reviews on the effects of EMFs on human health [1,2].

Wireless power transfer (WPT) can be utilized to supply power to equipment, and eliminates the need for a direct connection to a power source. Many experiments on WPT technology, such as laser, microwave and magnetic induction, have been carried out [3]. In recent years, Kurs *et al.* [4] and Karalis *et al.* [5] described a new EMF-related WPT technology using the resonant coupling phenomenon. This new WPT technology has many potential applications, such as wireless powering of residential and industrial equipment and wireless charging for electric vehicles, and has attracted the attention of many researchers who have started investigating related technologies [3,6]. Some studies have already discussed the possible relationship between EMFs from WPT using the resonant coupling phenomenon and human health based on the International Commission on Non-Ionizing Radiation Protection (ICNIRP) guidelines [7,8,9].

Evaluation of potential carcinogenesis at the cellular level requires assessment of cellular genotoxicity. The genotoxic effects of extremely low frequency (ELF) and radio frequency (RF) fields have been evaluated widely at the cellular level [1,2,10,11]. To our knowledge, however, there have been very few *in vitro* studies evaluating the genotoxic effects of fields near the 10-MHz frequency range. Therefore, in this study, we focused on the genotoxic effects for initial evaluation of the biological effects, and we evaluated the cell growth, cell cycle distribution, DNA strand breaks using the comet assay, micronucleus formation and hypoxanthine–guanine phosphoribosyltransferase (HPRT) gene mutation, using our new *in vitro* exposure system.

## 2. Experimental Section

### 2.1. Exposure System

**Figure 1:**
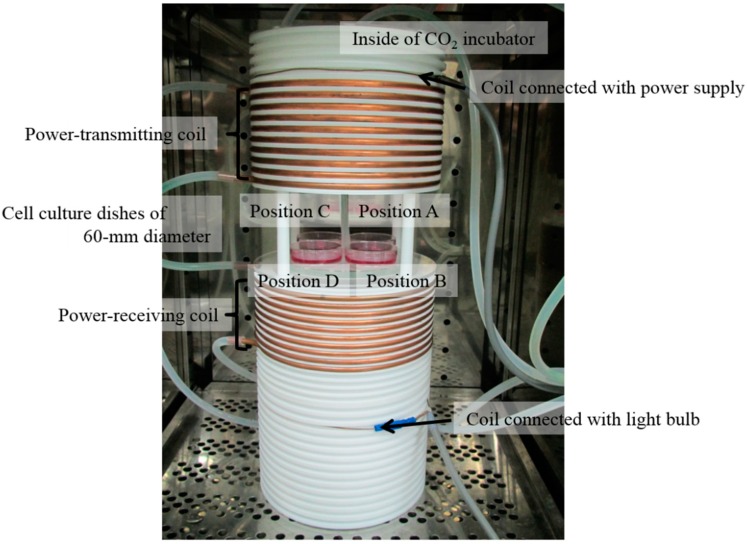
Photograph of coils designed for magnetic resonant coupling WPT and cell culture dishes of 60-mm diameter inside of the CO_2_ incubator.

WPT exposure was performed using our new *in vitro* exposure system with magnetic resonant coupling WPT. The details of our exposure system have been described previously [12]. In brief, the exposure system consists of coils designed for magnetic resonant coupling WPT built into a CO_2_ incubator (Model BNA-111, ESPEC, Osaka, Japan), a high-frequency power supply (Model T161-5356AEM, THAMWAY, Shizuoka, Japan), a thermometer (PalmSENSE, Photon Control, Burnaby, Canada) to measure the temperature of the cell culture medium, a water bath (Model SA-100, SANSYO, Tokyo, Japan) to maintain the cell culture medium at 37 °C ± 0.2 °C, and a light bulb. Magnetic resonant coupling WPT is performed with 85.4% power transfer efficiency at 200-W input power supply and 12.5-MHz resonant frequency. Four cell culture dishes of 60-mm diameter are placed between the power-transmitting coil and power-receiving coil, as shown in Figure 1. These coils are fixed tightly in grooves to avoid vibration caused by electromagnetic forces. The atmosphere in the CO_2_ incubator was maintained with humidified 95% air and 5% CO_2_. The restriction of magnetic fields and specific absorption rate (SAR) for workers, as stated in the ICNIRP guidelines [13,14], are 80 A/m and 20 W/kg (localized SAR (limbs)) at 10 MHz. The magnetic field and the SAR at each cell culture position in the exposure system, simulated using the finite element method (FEM) in a commercially available simulation platform (HFSS version 13.0.2, ANSOFT, Canonsburg, PA, USA) with a mesh size of 0.1 mm × 0.1 mm, are shown in Table 1. The control condition was compared with WPT exposure using a conventional CO_2_ incubator. Vibration of the exposure system and the conventional CO_2_ incubator for the control condition could not be detected by touch. The ambient magnetic fields inside the CO_2_ incubators were less than 0.55 μT (40 Hz–1 kHz), 0.13 μT (9 kHz–1 MHz), and 0.02 μT (1–30 MHz) measured using a magnetic field meter (TMM-2, Denryoku Techno Systems, Kawasaki, Japan; EHP-200A, Narda STS, Pfullingen, Germany), and less than 0.13 μT (40 Hz–1 kHz), 0.13 μT (9 kHz–1 MHz), and 0.02 μT (1–30 MHz) in our laboratory.

**Table 1:** Magnetic fields and SAR at each cell culture position. The data presented are expressed as mean ± standard deviation (SD).

Type	Position A	Position B	Position C	Position D
Magnetic	169.2 ± 2.5 A/m	170.7 ± 2.2 A/m	167.6 ± 1.7 A/m	171.5 ± 2.2 A/m
Field	(±1.5%)	(±1.3%)	(±1.0%)	(±1.3%)
SAR	21.8 ± 9.5 W/kg	21.3 ± 10.0 W/kg	21.6 ± 12.1 W/kg	20.7 ± 9.7 W/kg

### 2.2. Cells and Culture Conditions

Human embryo lung-derived SV40 virus transformed WI38VA13 subcloned 2RA cells (obtained from the Japanese Cancer Research Resources Bank, Osaka, Japan) were cultured in Eagle’s Minimum Essential Medium with L-glutamine and phenol red (Wako Pure Chemical Industries, Osaka, Japan) and 10% fetal bovine serum (Biowest, Nuaillé, France) at 37 °C in 95% air and 5% CO_2_.

### 2.3. Cell Growth

The WI38VA13 subcloned 2RA cells were seeded at a density of 2 × 10^5^ cells per dish, and were WPT exposed for 48, 96, or 144 h, respectively. The cells were then collected using trypsin-EDTA solution (25300, Thermo Fisher Scientific, Waltham, MA, USA) and counted with a particle counter (Model Z1, Beckman Coulter, Brea, CA, USA).

### 2.4. Cell Cycle Distribution

After exposure to WPT for 48, 96, or 144 h, cells were collected using trypsin-EDTA solution and fixed in 70% cold ethanol overnight at −20°C. The fixed cells were treated with 30 mg/mL Ribonuclease A (Sigma-Aldrich, St. Louis, MO, USA) for 30 min at room temperature and then stained with 1 mg/mL propidium iodide (PI) (P3566, Invitrogen, Carlsbad, CA, USA) for 10 min in an ice box. Cell cycle distribution was analyzed with a flow cytometer (FACS Calibur, Becton Dickinson, Franklin Lakes, NJ, USA). The cell cycle parameters from 10,000 gated nuclei were determined by CellQuest software. Ten thousand cells were analyzed per sample.

### 2.5. Comet Assay

A comet assay was performed using a Comet Assay Reagent Kit (4250-050-K, Trevigen, Gaithersburg, MD, USA), which contains glass slides, low melting agarose, lysis solution, 200 mM EDTA (pH 10), and SYBR Green (4250-050-05, Trevigen) as a fluorescent dye. Cells were seeded at a density of 1 × 10^6^ cells per dish. After incubation for 24 h in the conventional CO_2_ incubator, the cells were WPT exposed for 2 or 24 h and subsequently suspended (1 × 10^5^ cells/mL) in cold phosphate-buffered saline (PBS) (T900, Takara Bio, Shiga, Japan) on ice and mixed with molten agarose at 42 °C (cell suspension/agarose 1:10). Positive control cells were treated with 50 μg/mL bleomycin (B8416-15UN, Sigma-Aldrich) for 2 or 24 h. The cell suspension/agarose mixture (50 μL) was immediately pipetted onto a slide in the dark and left for 10 min at 4 °C for fixation, and then immersed in a lysis solution for 60 min at 4 °C. The slide was then immersed in Alkaline Unwinding Solution (NaOH pellets 0.6 g, 200 mM EDTA 250 μL, dH_2_O 49.75 mL per 50 mL) for 60 min at 4 °C in the dark. Electrophoresis was performed at 0.8 V/cm for 30 min at 4 °C, using Alkaline Electrophoresis Solution (NaOH pellets 12 g, 500 mM EDTA (pH 8) 2 mL, dH_2_O 1 L) as electrophoresis buffer. After gentle immersion twice in dH_2_O for 5 min each, fixation in 70% ethanol for 5 min, and drying at 37 °C for 15 min, the agarose including the cells was stained with 100 μL SYBR solution (SYBR Green (10,000× concentrate in DMSO) 1 μL, TE Buffer (pH 7.4) 10 mL) for 30 min at room temperature in the dark. The slide was then tapped gently to remove excess SYBR solution and rinsed briefly in water. The slide was dried overnight at 37 °C.

DNA strand breaks were analyzed using a Comet Assay IV (Perceptive Instruments, Bury St. Edmunds, UK), which includes software for calculation of DNA damage from images obtained in the comet assay. At least 300 comets from each of three replicate cultures (total: 900 comets) were analyzed.

### 2.6. Micronucleus Formation

Similarly to the comet assay, cells first incubated in the conventional CO_2_ incubator for 24 h were then WPT exposed for 2 or 24 h. Positive control cells were treated with 10 μg/mL bleomycin for 2 or 24 h. After exposure, cells were incubated with 3 μg/mL cytochalasin B (C2743-200UL, Sigma-Aldrich) in the conventional CO_2_ incubator for 24 h. The cells were collected at a concentration of 2.7 × 10^5^ cells/mL in PBS, and 0.1 mL of the samples was centrifuged onto slides using a centrifuge (Cytospin3, Thermo Fisher Scientific) at 900 rpm (91.45 g) for 5 min. The slides were then fixed with 80% cold ethanol for 30 min, washed lightly with PBS, and then soaked in new PBS for 5 min to completely remove the ethanol. Twenty microliters of PI (a final concentration of 0.2 μg/mL) diluted in dH_2_O was dropped onto the slides, which were then kept in the dark until counting was performed. A total of 1000 binucleated cells were scored for evaluation of the frequency of micronucleus formation, using fluorescence microscopy (AX70, Olympus, Tokyo, Japan).

### 2.7. HPRT Gene Mutation

The WI38VA13 subcloned 2RA cells were cultured with HAT supplemented medium (sodium hypoxanthine 100 μM, aminopterin 0.4 µM, and thymidine 16 μM) for 4 days. After the HAT treatment, cells were WPT exposed for 2 or 24 h after incubation in the conventional CO_2_ incubator for 24 h. Positive control cells were treated with 10 μg/mL bleomycin for 2 h. After exposure, the cells were washed with PBS three times and cultured in a conventional CO_2_ incubator for 24 h with new medium. During the culture period, mutations derived from DNA damage were fixed in the cells. After this period, the cells were re-seeded in 75-cm^2^ flasks and cultured for 4 days.

For selection of clones that are resistant to 6-thioguanine (6-TG), the cells (5 × 10^4^ cells) were plated in 10 dishes of 60-mm diameter containing medium with 15 µM of 6-TG (A4660, Sigma-Aldrich). Approximately 300 cells were plated in another 10 dishes of 60-mm diameter to determine the cloning efficiency. After 10 days of growth for the selection of cloning efficiency, or 11 days for the selection of clones, the number of colonies was scored.

### 2.8. Statistical Analysis

All experiments were repeated three times on separate days, and the data are expressed as the mean ± standard deviation (SD). Statistical analysis was conducted using Tukey’s test for multiple comparisons. P values less than 0.05 were considered statistically significant.

## 3. Results

### 3.1. Cell Growth

**Figure 2:**
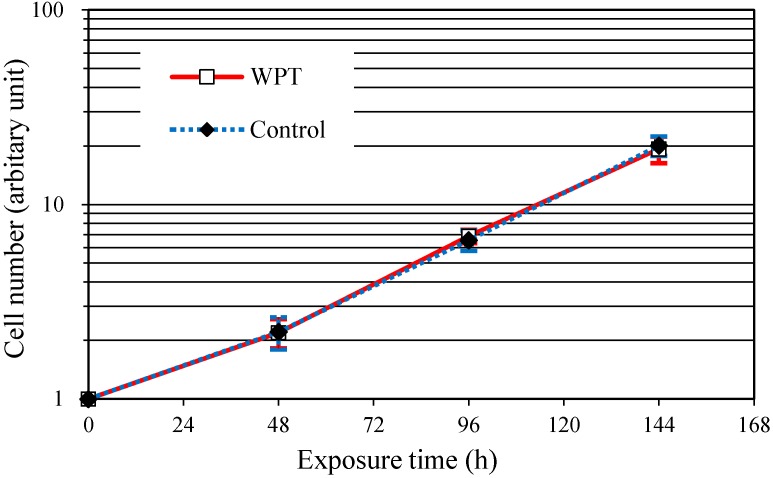
The growth curves for the WI38VA13 subcloned 2RA cells WPT exposed or control cells. The data presented are expressed as mean ± standard deviation (SD) of three independent experiments.

The growth curves of the WI38VA13 subcloned 2RA cells WPT exposed or control cells are shown in Figure 2. No significant difference in growth rate was observed for WPT-exposed cells and control cells.

### 3.2. Cell Cycle Distribution

**Figure 3:**
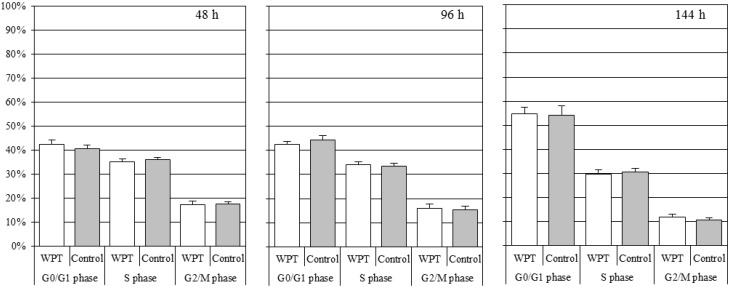
The cell cycle distribution for the WI38VA13 subcloned 2RA cells WPT exposed or control cells. White bars represent the WPT-exposed cells and gray bars represent the control cells. The data presented are expressed as mean ± standard deviation (SD) of three independent experiments.

Figure 3 shows the cell cycle distribution of the WI38VA13 subcloned 2RA cells WPT exposed or control cells. There was no significant difference in the cell cycle distribution between the WPT-exposed cells and the control cells.

### 3.3. Comet Assay

**Figure 4:**
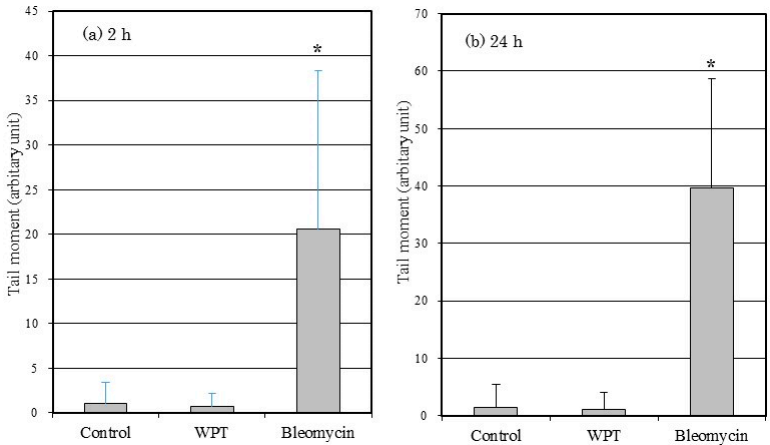
Tail moment in WI38VA13 subcloned 2RA cells WPT exposed, control cells, or treated with 50 μg/mL bleomycin for (**a**) 2 h or (**b**) 24 h. A total of 900 comets were scored. Data are presented as means ± SD from three separate experiments. * *p* < 0.05, compared with control. (Tail moment is defined as the product of the tail length and the fraction of total DNA in the tail.)

Figure 4 shows the tail moment of the WI38VA13 subcloned 2RA cells WPT exposed or control cells. No statistically significant difference was observed between the WPT-exposed cells and the control cells. The tail moment of the cells treated with bleomycin differed significantly compared with control cells and with WPT-exposed cells.

### 3.4. Micronucleus Formation

**Figure 5:**
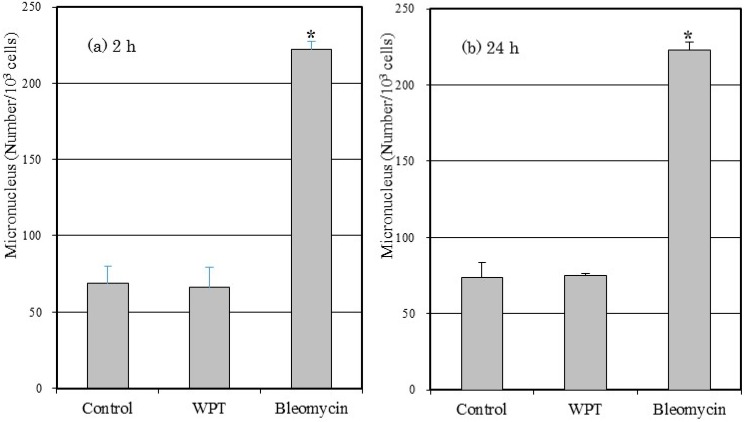
Numbers of induced micronuclei formation in the WI38VA13 subcloned 2RA cells WPT exposed or control cells, or treated with 10 μg/mL bleomycin for (**a**) 2 h or (**b**) 24 h. A total of 1000 binucleated cells were scored. Data are presented as means ± SD from three separate experiments. * *p* < 0.05, compared with control.

The frequencies of micronucleus formation in the WI38VA13 subcloned 2RA cells WPT exposed or control cells are shown in Figure 5. There was no statistically significant difference in micronucleus formation in WPT-exposed cells compared to control cells. The frequency of micronucleus formation in cells treated with bleomycin differed significantly compared with control cells and with WPT-exposed cells.

### 3.5. Mutation of the HPRT Gene

**Figure 6:**
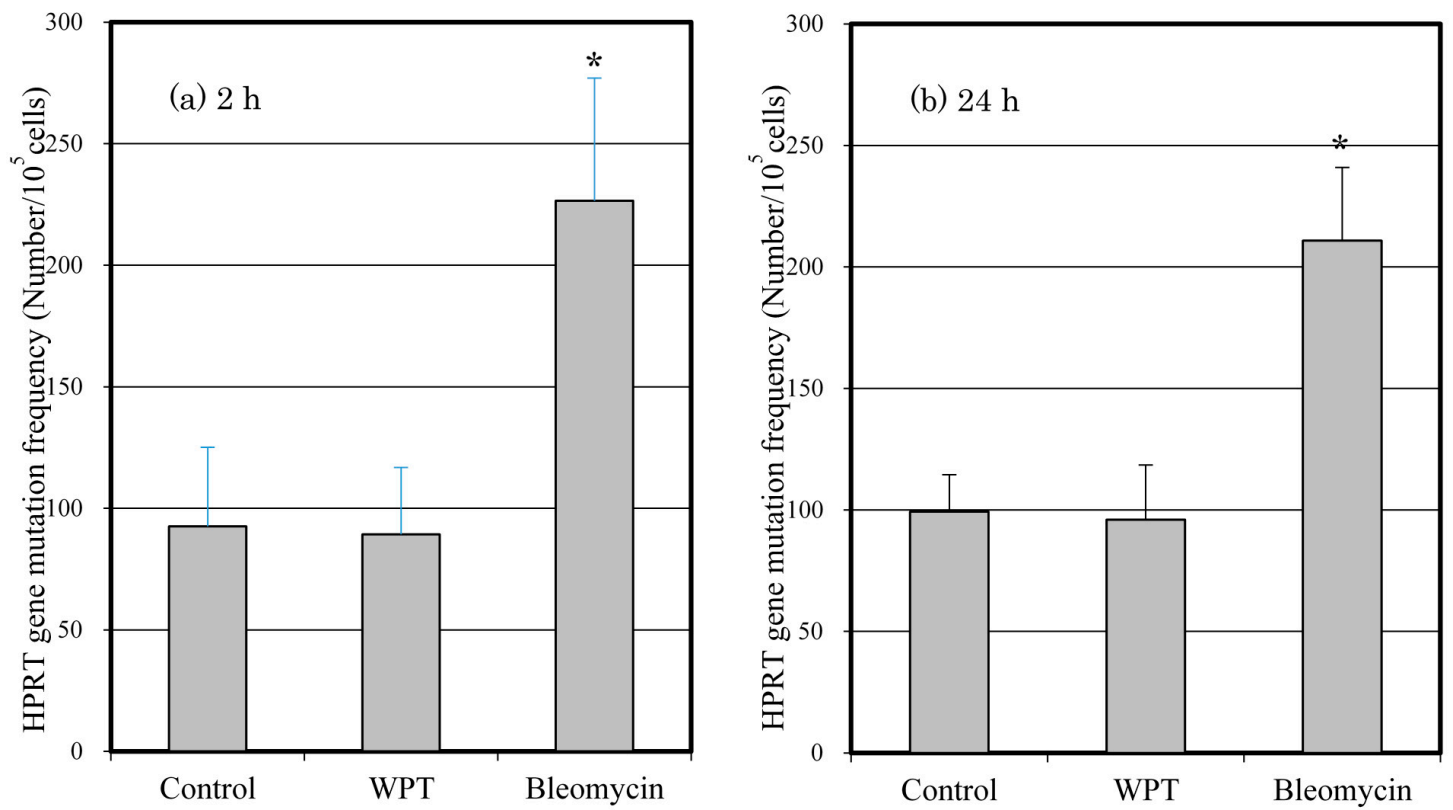
The frequency of HPRT gene mutation in the WI38VA13 subcloned 2RA cells WPT exposed or control cells for (**a**) 2 h or (**b**) 24 h, or treated with 10 μg/mL bleomycin for 2 h. Data are presented as means ± SD from three separate experiments. * *p* < 0.05, compared with control.

The mutation frequencies of the WI38VA13 subcloned 2RA cells WPT exposed or control cells are shown in Figure 6. There was no statistically significant difference in mutation frequency of WPT-exposed cells compared to control cells. The mutation frequency of cells treated with bleomycin differed significantly compared with control cells and with WPT-exposed cells.

## 4. Discussion

To investigate whether exposure to magnetic resonant coupling WPT has genotoxic effects on WI38VA13 subcloned 2RA cells, we first evaluated cell growth and cell cycle distribution under the WPT exposure condition for 48, 96, or 144 h, respectively. Genotoxic damage may affect the cell growth rate and cell cycle distribution through phenomena such as apoptosis and cell cycle arrest. In addition, the possibility of weak artifacts such as vibration is a concern during cell culture. However, as shown in Figure 2 and Figure 3, there were no significant differences between WPT-exposed cells and control cells. These results suggested that WPT exposure did not affect the cell growth rate and cell cycle distribution and that the affect from vibration of the exposure system was negligible.

We then performed a comet assay to evaluate DNA strand breaks after WPT exposure for 2 or 24 h. A comet assay is a sensitive and rapid method for detection of DNA strand breaks in individual cells. The principle for detecting DNA strand breaks is based on the ability of denatured, cleaved DNA fragments to migrate out of the nucleus under the influence of an electric field. When an electric field is applied to the DNA, undamaged DNA strands remain within the nucleus, whereas fragments from the smaller, broken strands of DNA migrate out of the nucleus towards the anode. Consequently, the comet pattern with a circular head and a tail (consisting of undamaged DNA and damaged DNA, respectively) is generated. The longer length and higher fluorescence intensity of the tail indicate a higher level of DNA damage [15]. As shown in Figure 4, cells treated with bleomycin alone, as a positive control, show statistically significant differences in tail moment compared with control cells. However, WPT-exposed cells show no statistically significant differences compared with control cells. Therefore, we conclude that WPT exposure did not cause any detectable DNA strand breaks.

We subsequently performed a micronucleus formation assay to evaluate chromosomal aberration after WPT exposure for 2 or 24 h. A micronucleus formation assay is a method to evaluate chromosomal aberration via detection of micronuclei in the cytoplasm of interphase cells. Micronuclei may originate from acentric fragments or whole chromosomes that are unable to migrate to the main nuclei during the anaphase stage of cell division [16]. As shown in Figure 5, we detected statistically significant differences in micronucleus formation of bleomycin-treated cells compared with control cells, but did not detect statistically significant differences in micronucleus formation of WPT-exposed cells compared with control cells. Therefore, we conclude that WPT exposure did not cause any detectable chromosomal aberration.

Finally, we performed an HPRT gene mutation assay after WPT exposure for 2 or 24 h. The HPRT gene mutation assay is a method for selecting clones that are resistant to the cytotoxic effects of 6-TG. Cells deficient in the HPRT gene due to gene mutation are resistant to 6-TG. HPRT gene-proficient cells are sensitive to 6-TG, which inhibits cellular metabolism and halts further cell division. Thus, mutant cells are able to proliferate in the presence of 6-TG, whereas normal cells, which contain the HPRT gene, are not able to proliferate [17]. Consistent with results of the comet assay and micronucleus formation, we detected a statistically significant increase in the HPRT gene mutation frequency of bleomycin-treated cells, as shown in Figure 6, but WPT exposure did not affect the mutation frequency, which demonstrated that the exposure conditions used in this study did not cause any detectable HPRT gene mutation.

## 5. Conclusions

We investigated whether exposure to magnetic resonant coupling WPT has genotoxic effects on WI38VA13 subcloned 2RA cells by examining cell growth, cell cycle distribution, DNA strand breaks, micronucleus formation, and HPRT gene mutations. We did not detect any effects between WPT-exposed cells and control cells. Our results suggest that WPT exposure under the conditions of the ICNIRP guidelines does not cause detectable cellular genotoxicity in human fibroblast cells. We are currently planning to investigate other cellular functions to further elucidate the possible health effects of WPT exposure.
